# Practical guidance for echocardiography for cancer therapeutics-related cardiac dysfunction

**DOI:** 10.1007/s12574-020-00502-9

**Published:** 2020-11-07

**Authors:** Tetsuari Onishi, Yuko Fukuda, Sakiko Miyazaki, Hirotsugu Yamada, Hidekazu Tanaka, Jiro Sakamoto, Masao Daimon, Chisato Izumi, Akiko Nonaka, Satoshi Nakatani, Makoto Akaishi, Guideline Committee of the Japanese Society of Echocardiography

**Affiliations:** 1 Hyogo Cardiovascular Center, Himeji, Japan; 2 Hyogo Cancer Center, Akashi, Japan; 3 Juntendo University, Tokyo, Japan; 4 Tokushima University, Tokushima, Japan; 5 Kobe University, Kobe, Japan; 6 Tenri Hospital, Tenri, Japan; 7 University of Tokyo, Tokyo, Japan; 8 Department of Cardiovascular Medicine, National Cerebral and Cardiovascular Center, 6-1 Kishibeshimmachi, Suita, Osaka Japan; 9 Saiseikai Senri Hospital, Suita, Japan; 10 Well-Aging Kyobashi Cardiovascular Clinic, Tokyo, Japan

**Keywords:** Cancer, Cardiomyopathy, Ejection fraction, Longitudinal strain, Chemotherapy

## Abstract

The prognosis of patients with cancer has improved due to an early diagnosis of cancer and advances in cancer treatment. There are emerging reports on cardiotoxicity in cancer treatment and on cardiovascular disease in cancer patients, from which cardiovascular disease has been recognized as a common cause of death among cancer survivors. This situation has led to the need for a medical system in which oncologists and cardiologists work together to treat patients. With the growing importance of onco-cardiology, the role of echocardiography in cancer care is rapidly expanding, but at present, the practice of echocardiography in clinical settings varies from institution to institution, and is empirical with no established systematic guidance. In view of these circumstances, we thought that brief guidance for clinical application was necessary and have therefore developed this guidance, although evidence in this field is still insufficient.

## Cancer therapeutics-related cardiac dysfunction: general

### Background

The prognosis of patients with cancer has improved due to an early diagnosis of cancer and advances in cancer treatment, while the number of cancer patients tends to increase over time with the aging of society [1–3]. There are emerging reports on cardiotoxicity in cancer treatment and on cardiovascular diseases in cancer patients, from which cardiovascular disease has been recognized as a common cause of death among cancer survivors [4–7]. 

In the meantime, patients with heart failure are reported to have increased risk of cancer [8]. Indeed, cancer is the leading cause of death, and cardiovascular disease is the second leading cause of death in Japan, with increasing numbers of patients suffering from both conditions due to the aging of society and advances in cancer treatment. This situation has led to the need for a medical system in which oncologists and cardiologists work together to treat patients.

With the growing importance of onco-cardiology, the role of echocardiography in cancer care is rapidly expanding, but at present, the practice of echocardiography in clinical settings varies from institution to institution, and is empirical with no established systematic guidance. In view of these circumstances, we thought that brief guidance for clinical application was necessary and have, therefore, developed this guidance, although evidence in this field is still insufficient.

### Definition of terms “cancer therapeutics-related cardiac dysfunction”

In general, cardiovascular complications of cancer therapy can be divided into the following nine categories [9]: (1) myocardial dysfunction and heart failure, (2) coronary artery disease, (3) valvular heart disease, (4) arrhythmias, especially those induced by QT-prolonging drugs, (5) arterial hypertension, (6) thromboembolic disease, (7) peripheral vascular disease and stroke, (8) pulmonary hypertension, and (9) pericardial complications.

Cancer therapeutics-related cardiac dysfunction refers primarily to (1) myocardial dysfunction and heart failure. This condition is typically abbreviated as “CTRCD,” but the definition of CTRCD is inconsistent and also may refer to ChemoTherapy-related Cardiac Dysfunction. Accordingly, to avoid confusion, the term “CTRCD” is not used in this guidance.

This guidance is primarily concerned with the clinical practice of echocardiography for chemotherapy-related cardiac dysfunction. Cancer treatment-related pulmonary hypertension, cancer-related thrombosis, and radiation-induced heart disease are also described briefly. The term “radiation-induced heart disease (RIHD)” is used distinctly from chemotherapy-related cardiac dysfunction.

### Classification of cancer therapeutics-related cardiac dysfunction (Table 1)

**Table 1:** Classification and characteristics of anticancer therapeutics-related cardiac dysfunction

	Type I	Type II
Characteristic agents	Anthracyclines (doxorubicin, daunorubicin, epirubicin, liposomal doxorubicin, mitoxantrone)	Anti-HER2 antibody (trastuzumab) Tyrosine kinase inhibitors (sunitinib, imatinib, lapatinib, osimertinib)
Mechanism of cardiac dysfunction	Direct cardiomyocyte necrosis Free radical formation Oxidative stress/damage	Cardiomyocyte dysfunction Blocked ErbB2 signaling
Natural course, clinical course, and response to treatment	Permanent cardiomyocyte damage, occurring from the initial administration of the drug Persistent and irreversible cardiac dysfunction, and may stabilize	Typically, reversible cardiomyocyte dysfunction, good prognosis, high likelihood of recovery in 2–4 months
Myocardial biopsy	Vacuoles Myofibrillar disarray Necrosis (Ultrastructural abnormalities)	Endocardial changes reported No characteristic changes observed with Type I drugs (No apparent ultrastructural abnormalities)
Dose effects	Cumulative, dose-related	Not cumulative or dose related
Risk factors	Any condition that causes myocardial damage or increases load Genetic susceptibility to causative agents	Trastuzumab: recent use of anthracyclines Sunitinib: arterial hypertension Imatinib: fluid retention A wide variation in genetic susceptibility to among causative agents

Adopted and modified from Refs. [4, 11]

Anthracyclines directly induce myocardial damage and necrosis through oxidative stress and other mechanisms. Because of disability to repair or regenerate, cardiomyocytes are “irreversibly" injured, progressing to cardiotoxicity. Anthracycline-induced cardiotoxicity is dose dependent and even increases exponentially with dose [4].

In contrast, trastuzumab, an anti-HER2 antibody, can cause cardiomyocyte dysfunction but not necrosis, and its induced cardiotoxicity is reversible. Subsequently emerging tyrosine kinase inhibitors, such as sunitinib, imatinib, and sorafenib, which are also known as angiogenesis inhibitors because of their inhibitory action on vascular endothelial growth factor (VEGF) receptors, can cause reversible and dose-independent myocardial dysfunction but not necrosis [10].

Anthracycline-induced irreversible cardiac dysfunction is defined as Type I, while reversible cardiac dysfunction is defined as Type II [11]. However, about 20% of drugs classified as Type II may induce irreversible damage due to mechanisms overlapping with those of Type I drugs. In the actual treatment of cancer, many patients receive a combination of Types I and II drugs, with many cases of both types of cardiotoxicity.

Based on the concept that consequent cardiotoxicity and cardiac dysfunction are more important than the mechanisms by which drugs cause cardiotoxicity (irreversible or reversible, namely, Type I or II), the most recent American Society of Clinical Oncology (ASCO) guidelines do not adopt the terms Type I or II.

There are also emerging reports on the cardiotoxic effect of immune checkpoint inhibitors (ICIs), such as nivolumab [12].

### Incidence and risk factors of cancer therapeutics-related cardiac dysfunction

In patients with cancer treated with regimens of trastuzumab alone or in combination with anthracyclines, the incidences of cardiotoxicity have been reported to be 4–27% for left ventricular (LV) dysfunction, and 0.4–16% for heart failure. A study of trastuzumab combined with an anthracycline showed an extremely high incidence rate of LV dysfunction (27%) and heart failure (16%) [13]. The other studies of combinations of trastuzumab and anthracyclines, in which the time difference between the start of chemotherapy and trastuzumab was 21–105 days, showed incidences of LV dysfunction of 4%–18.6%, and heart failure of 0.4–4% [4].

Although ICI-associated cardiac dysfunction is rare, with an incidence of approximately 1% [12, 14], deaths from fulminant myocarditis have been reported [10, 12, 15, 16]. ICI-related myocarditis occurred most frequently in the weeks after administration, but one case occurred as much as one year after administration. Risk factors for ICI-related myocarditis include combination ICI therapy, current or history of concurrent use of anticancer drugs with strong cardiotoxicity, such as VEGF inhibitors, ICI-related skeletal myositis, underlying cardiovascular disease with previous myocardial injury, underlying autoimmune disease, and antibodies to self cardiac antigens expressed in tumors. ICI-associated myocarditis is treated with high-dose steroids, although some cases are fatal.

Risk factors for cancer therapeutics-related cardiac dysfunction have been described in published guidelines and position papers in the cardiovascular and oncological fields [9, 17–19]. Table 2 summarizes the key risk factors from these references.

**Table 2:** Risk factors of cancer therapeutics-related cardiac dysfunction

Cancer treatment-related risk factors	Patient background-related risk factors
High-dose anthracyclines (≥ 250 mg/m^2^ doxorubicin, ≥ 600 mg/m^2^ epirubicin) HER2 inhibitors (e.g., trastuzumab) VEGFR inhibitors (e.g., pazopanib) BRAF inhibitors (e.g., dabrafenib) Immune checkpoint inhibitors	Age > 65 years or < 15 years Pre-existing coronary artery disease History of chronic heart failure or cardiomyopathy At least two comorbidities among hypertension, diabetes mellitus, smoking, and obesity Previous or concomitant anthracycline treatment

### Prognosis and significance of early diagnosis of cancer therapeutics-related cardiac dysfunction

Anthracycline-related cardiotoxicity is classified into acute and chronic types according to the time of onset. Acute cardiotoxicity occurs shortly after drug administration, but it is very rare and usually reversible. Chronic cardiotoxicity is further divided into early-onset type, occurring within the first year of administration, and late-onset type, occurring several years after administration. Most studies of anthracycline-related cardiotoxicity were conducted retrospectively and showed highly inconsistent incidences and prognosis partly due to the widely varying definitions of cardiotoxicity and duration of follow-up [20]. Older studies reported that the 2-year survival rate was < 50% when anthracycline-related heart failure occurred [21, 22]. Felker et al*. *[23] reported that patients with cardiomyopathy due to doxorubicin had a poorer prognosis than those with idiopathic cardiomyopathy and ischemic heart disease. A prospective study of 2625 patients receiving anthracycline-containing regimen [24], reported in 2015, showed that anthracycline-induced cardiotoxicity (defined as a reduction in left ventricular ejection fraction [LVEF] of > 10 percentage points from baseline and LVEF of < 50%) occurred in 9% of the patients, with a median time to onset of 3.5 months (98% of cases occurred in the first year after the end of treatment). Of these, 71% had improved cardiac function, and 11% had full recovery by initiating cardioprotective agents soon after the detection of cardiac dysfunction. These results suggested that anthracycline induces cardiac myocyte death (which is irreversible) but the residual cardiac function can be ameliorated by early treatment with cardioprotective agents, such as angiotensin-converting inhibitors (ACE inhibitors)/angiotensin receptor blockers (ARBs) and β-blockers. In addition, current advances in nonpharmacological therapy for severe heart failure, such as cardiac resynchronization therapy, transcatheter mitral repair, and mechanical circulatory support, may further help to achieve better prognosis of anthracycline-induced cardiotoxicity than before.

Cardiotoxicity caused by trastuzumab, a molecular targeting agent, is generally considered reversible with a favorable prognosis. Although trastuzumab monotherapy rarely induces cardiac dysfunction, cancer therapeutics-related cardiac dysfunction occurs more frequently when trastuzumab is administered combined with anthracyclines, paclitaxel, or cyclophosphamide [25]. Echocardiography monitoring of LV systolic function is crucial because trastuzumab-induced-cardiac dysfunction responds well to drugs for heart failure, such as ACE inhibitors/ARBs and β-blockers, and trastuzumab can be resumed in many cases [26].

## Diagnosis of cancer therapeutics-related cardiac dysfunction

### Biomarkers

(i) Implications for biomarker measurements
  The required role of biomarkers in cancer therapeutics-related cardiac dysfunction is stratification of cardiac risks and early detection of cardiac dysfunction. So far, studies of biomarkers have focused mainly on troponins, B-type natriuretic peptide (BNP), and N-Terminal/Pro-B-Type natriuretic peptide (NT-proBNP). The results from prospective studies with adequate sample sizes are limited, and the appropriate timing and frequency, and the optimal cutoff values of biomarker measurements by cancer type and anticancer agent, remain insufficiently clear. Nevertheless, the European and American guidelines recommend biomarker measurements before, during and after cancer treatment (moderate recommendation) [18, 27].
  The expert consensus of the European Association of Cardiovascular Imaging (EACVI) recommends troponin I (TnI) measurement in addition to echocardiographic indices [28]. Measuring BNP and Tn levels before starting treatment may be useful at least for risk assessment and evaluation of changes over time in cardiac function.
(ii) Troponins
  Cardiac troponin I (cTnI) and TnT (cTnT) are biomarkers specific to cardiac dysfunction, and, in particular, cTnI has been commonly used in studies of anthracycline-related cardiotoxicity. Recently, high-sensitivity troponin (hsTn) has been available, allowing the detection of trace levels of troponin. Cardinale et al. [29]. described the relationship between cTnI and high-dose chemotherapy for the first time in 2000. Their study included patients with a range of cancers (*n* = 204), including breast cancer, lymphomas, and ovarian cancer, who were receiving various chemotherapy regimens. TnI was measured before, immediately after, and 12, 24, 36, and 72 h after every single cycle of chemotherapy, while echocardiography was performed before the initiation and 1, 2, 3, 4, and 7 months after the end of chemotherapy. Patients in the positive TnI group showed a significantly more persistent reduction in LVEF. Cardinale et al. [30] also conducted a study of a larger number of cancer patients (*n* = 703), measured TnI soon after chemotherapy and 1 month later, and demonstrated that patients whose TnI tests were both positive had significantly reduced LVEF and more cardiovascular events. Furthermore, they conducted a study in patients with elevated TnI levels soon after receiving high-dose anthracycline-based chemotherapy (*n* = 114). The patients were randomized into the enalapril and control groups. The incidence of heart failure was significantly reduced in the enalapril group [31]. Another study group reported that the association between elevated Tn and cardiotoxicity was not significant. These conflicting results may be explained by differences in timing of Tn measurements and Tn assay methods and the small numbers of patients.
  Trastuzumab, a molecular-targeting agent, is used in patients with HER2-positive breast and gastric cancer. There are limited reports on the implications of Tn measurements in trastuzumab-related cardiotoxicity. Sawaya et al. [32] demonstrated that a combination of elevated hsTnI and reduced global longitudinal strain (GLS) predicted subsequent cardiotoxicity in patients with breast cancer treated with anthracyclines, taxanes and trastuzumab (*n* = 81). Ky et al. [33] showed that early elevation of hsTnI levels (at 3 months) was associated with subsequent cardiac dysfunction in patients with breast cancer who received regimens similar to those used by Sawaya et al.
  Cases of immune checkpoint inhibitor-related fulminant myocarditis have been reported, although they are rare. At present, the implications of monitoring Tn levels remain unclear in such cases.
(iii) BNP/NT-proBNP
  BNP/NT-proBNP is secreted in response to ventricular volume loading and wall stress. They are critical biomarkers in the assessment of heart failure in routine clinical practice. Among patients with active cancer who received high-dose chemotherapy (*n* = 52), those with persistently increased BNP/NT-proBNP levels had decreased LVEF 6–12 months after treatment [34]. Among cancer patients who received anthracyclines (*n* = 109), those who experienced cardiovascular events had a significantly higher BNP value before the cardiac event [35]. In contrast, BNP/NT-proBNP has not been demonstrated to predict the development of cardiotoxicity in patients with breast cancer [36]. Based on these findings, BNP/NT-proBNP is likely to be helpful for detecting cardiac dysfunction in the remote period after treatment, although it does not appear to be useful for early detection of cardiotoxicity. It should be noted that BNP/NT-proBNP is influenced by age, renal function, inflammation due to cancer, and the presence of arrhythmias, such as extrasystoles and atrial fibrillation.

### Echocardiography

Echocardiography is the most commonly used diagnostic imaging tool for the assessment of cardiac function before, during and after cancer treatment because this procedure is non-invasive, can be performed repeatedly due to the absence of radiation exposure, and is widely available in current clinical settings. This modality is not only used to assess LV and RV sizes and cardiac function (contractility and distensibility), but also frequently used to diagnose ischemic heart disease, organic cardiovascular diseases, such as valvular disease, macrovascular disease, pericardial disease, cardiac tumors (primary and metastatic), and to assess the severity of these diseases [37]. Thus, echocardiography is widely useful not only for the diagnosis and management of cancer therapeutics-related cardiac dysfunction, but also for the diagnosis of cardiac abnormalities in the onco-cardiology field.

#### LV contractility

- LVEF
  LVEF is used in the definition of cancer therapeutics-related cardiac dysfunction, and requires accurate quantitative assessment.
  1. Measurement method
    The method of discs is recommended, whereby end-systolic and end-diastolic LV volumes are measured by tracing endocardial borders in apical four- and two-chamber views by two-dimensional echocardiography. However, LVEF determined by this method poses the issue that reproducibility is not always good. How to address this issue is discussed later (“Drug-induced pulmonary artery hypertension and cancer-associated thrombosis (CAT)” section).
    The American Society of Echocardiography (ASE) and the European Society of Cardiovascular Imaging (EACVI) recommend determining LVEF by three-dimensional (3D) echocardiography [28]. Due to advances in ultrasound diagnostic machines and automated measurement techniques, 3D echocardiography allows us to obtain more accurate and reproducible LV volume measurements than those measured by the method of discs in patients with 3D images of good quality [38]. However, the 3D method has not achieved the recommended level for the assessment of cancer therapeutics-related cardiac dysfunction in routine clinical use for the following reasons: there are only a limited number of centers performing 3D echocardiography routinely, reliable measurements cannot be obtained in some patients due to image quality issues [39]; unlike the disk method, the normal value of LVEF by 3D echocardiography has not been widely published, and the cut-off value for cancer therapeutics-related cardiac dysfunction has not been well established [40].
    Patients with cancer therapeutics-related cardiac dysfunction typically have diffuse LV wall motion abnormalities, but LV regional wall motion abnormalities may occur with certain anticancer drugs that increase the risk of developing ischemic heart disease [41]. Considering the fact that LV wall motion does not always impaired uniformly and that accurate quantification of LVEF is critical for the assessment of cancer therapeutics-related cardiac dysfunction, determination of LVEF by the eyeball method or the Teichholz method using M-mode echocardiography is inadequate.
  2. Normal value
    Based on the lower limit of normal for LVEF of 55% in the 2005 ASE guidelines for chamber quantification [42], LVEF of 55% had been used as the diagnostic criteria for cancer therapeutics-related cardiac dysfunction. The guidelines updated in 2015 provide the lower limit of normal for LVEF at 53%, taking into account the results of further studies [40]. Since the position paper of the European Society of Cardiology (ESC) defines cancer therapeutics-related cardiac dysfunction as “a decrease in LVEF of > 10 percentage points from baseline, to a value below the lower limit of normal” [9]. However, there is an issue of a measurement error in LVEF and normal value of LVEF may also change according to studies. Considering these matters, we set the cut-off value of LVEF at 50%. Therefore, a diagnosis of cancer therapeutics-related cardiac dysfunction is made when a patient has “a decrease in LVEF of > 10 percentage points from baseline, and to a value of < 50%.” (E.g., LVEF 57% → 46% meets the criteria, whereas LVEF 57% → 49% or LVEF 65% → 54% does not meet the criteria.) There is a possibility that the cut-off value may change by accumulation of evidence in Onco-Cardiology, thus a focused update should be implemented. In addition, it is important that we should do the close follow-up considering the time course of LVEF and clinical parameters, even if the value is not fulfilled the definition above.
- Global longitudinal strain (GLS)
  The method of discs has a measurement error of approximately 10% for LVEF [43, 44]. This value is equal to a diagnostic criterion (10% decrease from baseline) for cancer therapeutics-related cardiac dysfunction, which poses the significant issue that small changes in the LVEF value may not necessarily represent true changes due to reproducibility issues. Recently, GLS using speckle-tracking echocardiography has become utilized. GLS is a more sensitive and reproducible indicator of cardiac dysfunction than LVEF, and its use is recommended by the European and American cardiovascular guidelines [9, 28] and the American Society of Oncology guidelines.
  In institutions where GLS measurements by speckle-tracking echocardiography are not feasible, mitral annular plane systolic excursion (MAPSE) calculated by M-mode echocardiography (a measure of left ventricular longitudinal function similar to GLS) or mitral annular systolic velocity (S’) by tissue pulsed Doppler echocardiography is recommended [28]. However, since, unlike GLS, the MAPSE and S’ do not have cut-off values for detecting the cardiotoxicity of anticancer drugs, interpretation of the results limited to that latent LV dysfunction is suspected if a significant decrease is noted compared with the previous or baseline value.
  1. Measurement method
    GLS is measured using two-dimensional speckle-tracking echocardiography on three apical views (long-axis and two- and four-chamber views) from video data of cardiac cycles, using analysis software built into the device or on a computer. GLS is defined as the average peak strain of 18 LV segments.
  2. Normal value
    Takigiku et al. have reported the normal range of LV GLS in Japanese subjects [45].
    Table 3 summarizes the prediction of adverse cardiac events by GLS measurements before cancer treatment [46–49]. The cut-off values for GLS used in these studies ranged from 16 to 19%. Given that 18% is most commonly used as the lower limit of normal for GLS, a value of less than 16% is considered high risk, and 16–18% as borderline.
  Negishi et al. [50] reported that 11% reduction in GLS over time predicted subsequent decrease in LVEF. In 2016 ESC Position paper [9], a relative percentage reduction of GLS of > 15% from baseline after cancer treatment (e.g., 25% → 21%, resulting in a relative percentage reduction of 16%, meets the criteria; but 25% → 22%, resulting in a relative percentage reduction 12%, does not meet the criteria) should be considered as a marker of early cardiotoxicity of anticancer drugs (i.e., subclinical LV cardiac dysfunction), even without a significant decrease in LVEF [9, 28]. On the other hand, relative percentage reductions of GLS of < 8% from baseline after cancer treatment appear not to be clinically meaningful (no cardiotoxicity of anticancer drugs or subclinical LV cardiac dysfunction) [28]. There are also ongoing clinical studies (e.g., SUCCOUR trial), and the cutoff values for these GLS may change in the future.
  Table 4 shows the echocardiographic definitions of cancer therapeutics-related cardiac dysfunction in the relevant guidelines.

**Table 3:** Prediction of adverse cardiac events by GLS before cancer treatment

References	Anthracycline Dose* (mg/m^2^)	Type of cancer	No. of patients No. of events Type of event	Software GLS cutoff value	Comments
Mousavi [46]	207 ± 99	Breast cancer and hematological cancer	158 12 Heart failure	TomTec 16%	Hazard ratio, 4.7
Rhea [47]	Anthracyclines in 58% of patients Chest radiotherapy in 26%	Hematological cancer and solid tumors	122 59 All-cause death	Vivid 7/Q 18%	
Ali [48]	217 (8–670)	Leukemia and malignant lymphoma	450 28 Heart failure/Cardiac death	TomTec 17.5%	AUC, 0.89
Hatazawa [49]	285 ± 107	Malignant lymphoma	73 10 Heart failure	QLAB version 10 19%	AUC, 0.77 Sensitivity, 60% Specificity, 87%

*Expressed as doxorubicin equivalents

**Table 4:** Echocardiographic definitions of cancer therapeutics-related cardiac dysfunction

	ASCO clinical practice guideline [18]	ESMO clinical guideline [27]	EACVI/ASE expert consensus [28]	ESC position paper [9]
Definition based on echocardiographic parameters	Reduction in LVEF of > 10 percentage points from baseline (cited from the ASE guidelines)	Reduction in LVEF of > 10 percentage points from baseline	Decrease in LVEF of > 10 percentage points from baseline, to a value < 53% of baseline. In comparable cases, a reduction of GLS of > 15% is considered significantly abnormal	Decrease in LVEF of > 10 percentage points from baseline, to a value below the lower limit of normal Reduction in GLS of > 15% compared with baseline

A reduction in LVEF of 10 percentage points represents an absolute change of 10 percentage points, while a reduction in GLS of 15% represents a 15% reduction relative to baseline

*ASCO* American Society of Clinical Oncology, *ESMO* European Society of Medical Oncology; *ASE* American Society of Echocardiography, *ESC* European Society of Cardiology, *EACVI* European Association of Cardiovascular Imaging

#### LV diastolic function

Data supporting the utility of LV diastolic function parameters for the diagnosis, follow-up, and prognostic prediction of cancer therapeutics-related cardiac dysfunction are sparse. Nevertheless, a routine systematic evaluation of LV diastolic function should be performed together with LV filling pressure according to the recommendations of the existing guidelines [51]. Caution should be exercised when *E*/*e*' is used to estimate the LV filling pressure, because the loading conditions are altered by the side effects (nausea, vomiting, diarrhea) of cancer treatment. Elevated LV filling pressure is suggestive of heart failure. Patients with such findings should be referred to a cardiologist even if they are asymptomatic.

#### Right ventricular (RV) function and pulmonary artery pressure

Data supporting the utility of RV function evaluation for the diagnosis of cancer therapeutics-related cardiac dysfunction are sparse. An assessment of RV contractility is critical when drugs with the risk of pulmonary arterial hypertension, including dasatinib and other tyrosine kinase inhibitors, are used, or when cancer-associated thrombosis is suspected. Echocardiography is also helpful in diagnosing a cancer-related disease called pulmonary tumor thrombotic microangiopathy (PTTM), which is characterized by a sudden elevation of pulmonary pressure.

Patients who have enlarged RV or decreased RV contractility, or those who are suspected of having pulmonary hypertension in the echocardiographic examination, should be referred to a cardiologist.

### Other imaging modalities

(i) Nuclear cardiology (myocardial scintigraphy)
  ECG-gated pooled cardiac imaging enables the measurement of LVEF, in which technetium-99 m-labeled red blood cells in the heart chamber are counted using a γ camera to synchronize image acquisition with the heart rate. This technique allows the monitoring of cardiac function during chemotherapy, [52] and is indicated as class IA in the ACC/AHA guidelines [53]. Its utility in the early detection of cardiotoxicity has been demonstrated [54]. This method has good reproducibility and is helpful where LVEF cannot be assessed by echocardiography, but has the disadvantages of exposure to radiation and high cost.
(ii) Cardiac MRI
  Currently, cardiac MRI is regarded as the gold standard for measurement of the LV volume and LVEF, which provides the most accurate LV volume [55]. Cardiac MRI can also evaluate myocardial properties not possible by echocardiography. Cardiac MRI may be useful, specifically in those with suboptimal echocardiography or discrepant results. It is reported that delayed contrast enhancement is often distributed in the middle layer of the myocardium on the lateral wall in cardiomyopathy caused by trastuzumab [56]. A hyper-intense signal suggestive of potential cardiac dysfunction has been demonstrated by T1 mapping in some cancer survivors after anthracycline-based chemotherapy [57].
  Cardiac MRI is safe, accurate, and reproducible with no radiation exposure, and is used for the diagnosis of cardiac involvement or metastasis of cancer, apart from cancer therapeutics-related cardiac dysfunction. However, this method has the disadvantages of being time-consuming and expensive, which limits the number of institutions that can implement it. Therefore, cardiac MRI should be considered when LVEF assessment by echocardiography is difficult.

## Echocardiographic protocols for patients treated with anticancer drugs

### Echocardiography before treatment with anticancer drugs (Tables 5, 6)

**Table 5:** Preferable frequency of follow-up echocardiography

	Before treatment	During treatment	After treatment
Anthracyclines	Mandatory	When the accumulating dose (*1) > 240 mg/m^2^ (*2) > 500 mg/m^2^ (*2) End of treatment	6 and 12 months after the end of treatment As needed thereafter (see Fig. 1; Tables 6, 8)
Anti-HER2 monoclonal antibodies	Mandatory	Every 3 months End of treatment	Finish the follow-up if there is no decrease in LVEF/GLS at the end of treatment
Molecular-targeting agents other than HER2	Mandatory	When clinically indicated, (*4) by referring to the description of the Guide for Proper Use of each drug (*3) End of treatment	Finish the follow-up if there is no decrease in LVEF/GLS at the end of treatment
Immune checkpoint inhibitors	Mandatory	End of treatment	Finish the follow-up if there is no decrease in LVEF/GLS at the end of treatment

(*1) Doses are expressed as doxorubicin equivalents (e.g., the incidence of epirubicin-related cardiotoxicity is 0.66 relative to that of doxorubicin, indicating that the dose 360 mg/m^2^ of epirubicin is approximately equipotent to 240 mg/m^2^ of doxorubicin)

(*2) If it is difficult to know for certain the doses of anticancer drugs, echocardiography may preferably be repeated about every 3 months during the follow-up period

(*3) Refer to Appendix Table 12

(*4) For example, when clinical manifestations appear, or the cardiac shadow appears to be enlarged on thorax XP or CT scans, compared with that before treatment

**Table 6:** Preferable duration of echocardiographic follow-up for typical anticancer drugs

	Trade name	Non-proprietary name (Abbreviation)	Recommended timing of echocardiography
Anthracyclines	Adriacin	Doxorubicin (DXR, ADM, ADR)	Before treatment, at accumulating doses of 240 and 500 mg/m^2^ and end of treatment, and 6 and 12 months after the end of treatment
	Therarubicin	Pirarubicin (THP)	Before treatment, at accumulating doses of 400 and 800 mg/m^2^ and end of treatment, and 6 and 12 months after the end of treatment
	Epirubicin, Farmorubicin	Epirubicin (EPI)	Before treatment, at accumulating doses of 360 and 900 mg/m^2^ and end of treatment, and 6 and 12 months after the end of treatment
	Doxil	Doxorubicin hydrochloride liposome formulation (PLD)	Before treatment, at accumulating doses of 240 and 500 mg/m^2^ and end of treatment, and 6 and 12 months after the end of treatment
Anti-HER2 monoclonal antibodies	Herceptin	Trastuzumab	Before treatment, every 3 months during treatment, and at the end of treatment
	Kadcyla	Trastuzumab emtansine	
Molecular-targeting agents other than HER2	Lembima	Lenvatinib	Before treatment, as clinically indicated* afterward, and at the end of treatment
	Votrient	Pazopanib	
	Tagrisso	Osimertinib	
	Tafinlar/Mekinist	dabrafenib/trametinib	
	Avastin	Bevacizumab	
	Sutent	Sunitinib	
	Tykerb	Lapatinib	
Immune checkpoint inhibitors	Yervoy	Ipilimumab	Before treatment and at the end of treatment
	Opdivo	Nivolumab	
	Keytruda	Pembrolizumab	
	Bavencio	Avelumab	
	Tecentriq	Atezolizumab	
	Imifinzi	Durvalumab	

^*^Refer to (*4) in Table 5

The purposes of echocardiographic examination prior to anticancer drug treatment are to assess cardiovascular risk, to predict possible cardiovascular complications, and to obtain control data for the early diagnosis of cardiovascular complications in the course of treatment. The examination should be conducted in all patients before initiating regimens containing drugs listed in Table 1 and immune checkpoint inhibitors.

An assessment of all routine echocardiographic parameters is mandatory. In particular, LVEF measurement is paramount, since this parameter is the basis of the definition of cancer therapeutics-related cardiac dysfunction. GLS is also recommended as a more sensitive indicator of left ventricular cardiac dysfunction, and GLS measurement is mandatory where feasible since this is recommended as a more sensitive parameter than LV cardiac dysfunction.

### Echocardiography during treatment with anticancer drugs

**Fig. 1:**
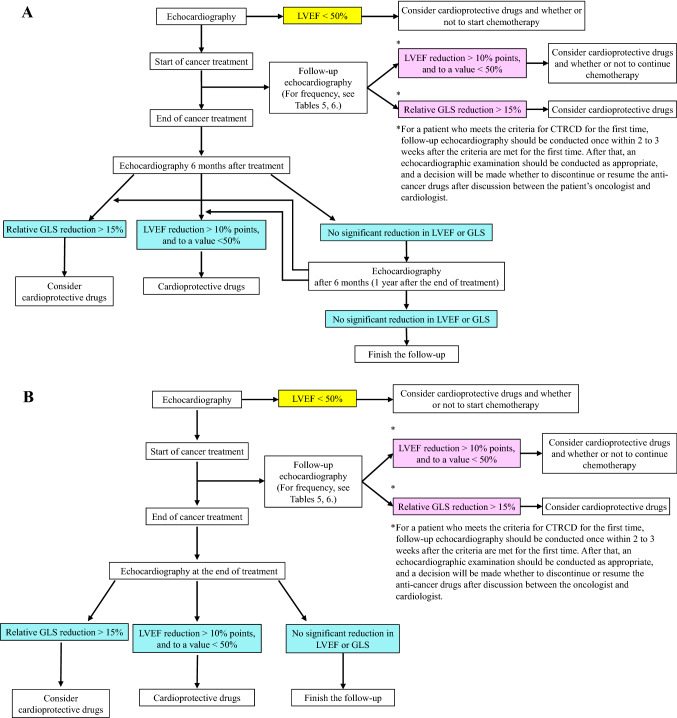
Frequency and parameters of the echocardiographic follow-up in treatment with anticancer drugs. (**a** patients who have received anthracyclines, **b** patients who have never received anthracyclines)

(i) Frequency of follow-up (Tables 5, 6; Fig. 1)
  Anthracycline-related cardiac dysfunction is dose related. In other guidelines, [58] echocardiographic imaging is recommended in patients receiving anthracyclines at cumulative doses of > 240 mg/m^2^, and follow-up echocardiography should be repeated by accumulating additional doses. Regarding timing, it is recommended to perform echocardiography before each additional dose of 50 mg/m^2^, but in clinical practice, it is difficult to perform a follow-up examination at this frequency. Therefore, after consultation with physicians involved in cancer treatment, we have decided that echocardiography should be performed when the accumulated dose exceeds 500 mg/m^2^ in this guideline. Anthracycline-based anticancer regimens are listed by cancer type in Appendix Table 11, together with doses expressed as adriamycin equivalents. In some patients, it is difficult for cardiologists to know for certain the appropriate doses of anticancer drugs. In such cases, echocardiography may preferably be repeated about every 3 months during the follow-up period, and the frequency may be determined according to the circumstances and conditions of each institution. Most importantly, the required follow-up echocardiography should not be missed.
  Cardiac dysfunction due to trastuzumab, an anti-HER2 monoclonal antibody, is not dose related. Follow-up evaluation every 3 months is desirable during treatment, as described in a Scientific Statement from American Heart Association [58]. Similar timing of follow-up evaluation is prescribed for novel anti-cancer drugs in their Guides for Proper Use. All of the recommended frequencies in these Guides for Proper Use have inadequate evidence, but are largely based on echocardiography schedules specified in the clinical study protocols of individual drugs. Therefore, it is not practical to perform echocardiography at every timing described in the Guides for Proper Use. In clinical practice use, echocardiographic evaluation should be considered as clinically indicated, for example, when clinical manifestations appear, or the cardiac shadow appears to enlarge on thorax XP or CT scans, compared with before treatment.
  For a patient who meets the criteria for cancer therapeutics-related cardiac dysfunction for the first time, his/her oncologist and cardiologist should discuss whether or not to use cardioprotective drugs and whether or not to continue the anticancer drugs. Follow-up echocardiography should be conducted once within 2–3 weeks after meeting the criteria for the first time. After that, an echocardiographic examination should be conducted as appropriate, and a decision will be made as to whether to resume or discontinue the anti-cancer drugs after discussion between the oncologist and the cardiologist.
  Follow-up intervals may be adjusted, taking into account the risk factors listed in Table 2.
  Table 5 lists the recommended frequency of follow-up echocardiography, and Table 6 shows typical anticancer drugs.
(ii) Evaluation parameters
  The aim of the imaging examination during treatment is the early detection of cancer therapeutics-related cardiac dysfunction to initiate the administration of cardioprotective agents and adjustment of the anticancer regimen to allow cancer treatment to be completed as far as possible. As described in the previous section, it is challenging to repeat echocardiography as frequently as recommended in all cancer patients during treatment, partly due to limited laboratory human resources.
  Therefore, mandatory items should be LV contractility, which is the basis of the definition of cancer therapeutics-related cardiac dysfunction, and other parameters that are helpful for the early detection of heart failure (Table 7) [28, 59, 60].
  Comparison with previous or baseline values is essential for any parameters.

**Table 7:** Echocardiographic parameters evaluated during treatment with anticancer drugs

Mandatory items	Possible Substitutes
LVEF by the disc method LV end-diastolic diameter/LV end-systolic diameter LV diastolic parameters (*E*/*A*, *E*/*e*ʹ) Tricuspid regurgitant velocity GLS (where feasible)	*s*ʹ and MAPSE (as a substitute for GLS)

*E* early diastolic transmitral flow velocity, *A* atrial systolic transmitral flow velocity, *e*ʹ early diastolic mitral annular velocity, *GLS* global longitudinal strain, *MAPSE* mitral annular plane systolic excursion, *s*ʹ mitral annular systolic velocity

### Echocardiography after treatment with anticancer drugs

1. Frequency and duration of follow-up (Tables 5, 6, 8; Fig. 1)
  Both pediatric and adult survivors of chemotherapy have a lifelong risk of development of cardiac dysfunction [9]. In particular, survivors treated with anthracyclines are more likely to develop cardiac dysfunction and require regular follow-up using echocardiography, biomarkers, and other measures. Since 98% of cases of anthracycline-induced cardiac dysfunction are reported to occur within the first year, with a mean time to development of 3.5 months, particularly close follow-up is necessary during the first 6 months of treatment [24].
  Although there is no evidence on the time of completion of follow-up, patients who have received cardioprotective agents or whose regimen has been modified or altered during and after cancer treatment because of reduced cardiac function should be followed up on for life (preferably about once a year but it depends on the individual cardiac function and symptoms). Among cancer survivors who have no abnormal cardiac function during and 6 months after treatment, those who have received anthracyclines should be followed up on for up to 1 year after treatment, and their follow-up may be finished if no abnormal cardiac function occurs by that time; whereas for those who have never received anthracyclines, the follow-up can be finished if there are no abnormalities in the examination at the end of treatment. The intervals and duration of follow-up echocardiography are determined as appropriate by referring to Table 2 of risk factors.
2. Evaluation parameters
  Follow-up examinations after the end of treatment should include all parameters evaluated in routine echocardiography, as with the examination before treatment. The same is true for GLS measurements.

**Table 8:** Recommended duration of the echocardiographic follow-up after treatment with anticancer drugs

Cancer survivors who have received cardioprotective agents or whose regimen has been modified or altered during and after treatment because of reduced cardiac function	Follow-up for life (preferably about once a year)
Cancer survivors who have received anthracyclines with no abnormal cardiac function during and 6 months after treatment	Finish the follow-up at 1 year after treatment if no abnormalities are observed then
Cancer survivors who have never received anthracyclines with no abnormal cardiac function during and at the end of treatment	Finish the follow-up at the end of treatment
Cancer survivors who have developed clinical manifestations and/or abnormalities in other examinations	Determine as appropriate

## Drug-induced pulmonary artery hypertension and cancer-associated thrombosis (CAT)

Although this guidance is mainly intended to help the practice of echocardiography in the medical care of cancer therapeutics-related cardiac dysfunction, cancer treatment-related pulmonary hypertension, cancer-associated thrombosis, and radiation-induced heart disease are briefly described in “Drug-induced pulmonary artery hypertension and cancer-associated thrombosis (CAT)” and “Radiation-induced heart disease (RIHD)” sections.

### Pulmonary hypertension

In the clinical classification of pulmonary hypertension, pulmonary hypertension induced by anticancer drugs is included in Group 1 as drug-induced pulmonary arterial hypertension (DPAH) [61]. Some alkylating agents (mitomycin C and cyclophosphamide) and interferon-α are traditionally considered possible risk factors of pulmonary hypertension. More recently, pulmonary hypertension has been reported in patients with chronic myeloid leukemia after treatment with dasatinib (tyrosine kinase inhibitor) [62, 63]. Dasatinib was considered a likely risk factor of pulmonary hypertension in the 2018 Nice Classification [64]. Of note, pulmonary hypertension due to chronic myeloid leukemia itself is classified as Group 5.

### Thrombosis

In 1865, Trousseau described the association between migratory thrombophlebitis and occult malignancy for the first time, [65] and in 1936, Gross and Friedberg reported that patients with cancer were more likely to develop nonbacterial thromboendocarditis (NBTE) [66]. Systemic embolism induced by cancer-related hypercoagulability or NBTE in patients with cancer is called “Trousseau's syndrome,” for which special attention is needed because thrombosis can occur in both veins and arteries.

Cancer cells produce some factors that activate the coagulation pathway such as tissue factor. In addition, patients with cancer may develop blood flow stasis due to decreased physical activity or compression of vessels due to a tumor, and vascular endothelial injury can be induced by surgery and chemotherapy. Thus, cancer patients often suffer from Virchow’s triad (hypercoagulability, blood flow stasis, and vascular endothelial injury), which is a risk factor of thromboembolism. Thromboembolism is the second leading cause of death, following progression of cancer, in cancer patients treated with chemotherapy [67].

Among cancer-associated thrombosis, cerebral infarction is sometimes specifically called "Trousseau's syndrome" in a narrow sense. NBTE, one of the causes of cerebral infarction, develops most commonly in patients with adenocarcinomas, including lung, pancreatic and gastric cancer among the types of cancer. If patients with cancer develop cerebral infarction, echocardiography should be performed to evaluate for the presence of NBTE. Heparin, but not warfarin, is often helpful for the treatment of NBTE.

The incidence of venous thromboembolism (VTE) tends to increase year by year in cancer patients but not in non-cancer patients [68]. Cancer is a major risk factor for VTE, and patients with cancer account for 23–27% of patients with VTE [69, 70]. Among the types of cancer, the incidence of VTE is higher in gynecologic or hematopoietic malignancies relative to the prevalence of these malignancies [70]. Patients with cancer often suffer from recurrent VTE or major bleeding due to difficulties with anticoagulation control. VTE is also more likely to develop in patients with cancer during chemotherapy and those with distant metastases [70]. Therefore, patients with cancer should be treated with awareness of the high risk of VTE according to cancer sites and cancer status. Low-molecular-weight heparin (LMWH) is not used for the treatment of VTE in standard clinical practice in Japan because this indication is not covered by the National Health Insurance, while LMWH is the standard therapy for cancer-associated VTE in Europe and the U.S. Warfarin is often less controllable than LMWH. Emerging evidence that direct oral anticoagulants (DOACs) are noninferior to LMWH for the treatment of VTE [71, 72] has been spurring the use of DOACs for this condition.

## Radiation-induced heart disease (RIHD)

Chest radiotherapy is used for the treatment of malignant lymphoma and breast, lung and esophageal cancer. It was once believed that radiation had minimal effect on the heart and blood vessels, but it is increasingly recognized that heart disease may develop as a late complication as long-term cancer survivors increase due to advances of cancer therapy. Radiation can cause pericarditis, cardiomyopathy, valvular disease, coronary artery disease, and carotid artery disease, depending on the area irradiated, with incidences of 10–30% in patients at 5–10 years after radiotherapy. RIHD, a heterogeneous disease that can manifest years or decades, is associated with high morbidity and mortality [73]. Patients with risk factors for RIHD, including younger age, high radiation doses, and use of anthracyclines (Table 9), require special caution [74].

**Table 9:** Risk factors for the development of RIHD

Anterior or left chest irradiation location
High cumulative dose of radiation (> 30 Gy)
Younger patients (< 50 years)
High dose of radiation fractions (> 2 Gy/day)
Presence and extent of tumor in or next to the heart
Lack of shielding
Concomitant chemotherapy (anthracyclines considerably increase the risk)
Cardiovascular risk factors (i.e., diabetes mellitus, smoking, overweight, hypertension, hypercholesterolemia)
Pre-existing cardiovascular disease

High risk is defined as an anterior or left chest irradiation location, plus one or more of the other risk factors listed above. Modified from Ref. [73]

### Common types of RIHD

(i) Pericardial disease
  Acute pericarditis is an early complication of radiotherapy. Its incidence has decreased by modern radiotherapy techniques, such as reduction in dose and field size [75]. Delayed pericarditis may manifest several weeks to years after radiotherapy, with pericardial fibrous thickening and adhesions, chronic constriction, and chronic pericardial effusion, progressing to constrictive pericarditis. Constrictive pericarditis can be observed in about 4–20% of patients, and its incidence increases with increasing radiation dose [74].
(ii) Cardiomyopathy
  Cardiomyocytes themselves are resistant to radiation because they do not undergo cell division, but the vascular endothelial injury can be induced by radiation. The resulting microvascular damage can cause ischemia and eventually myocardial dysfunction. Myocardial compliance decreases as myocardial fibrosis progresses, possibly leading mainly to diastolic dysfunction, although systolic dysfunction also may occur. Conduction disturbance may occur. Incidence of cardiomyopathy is reported about 10% [76]. In patients with Hodgkin lymphoma treated with non-anthracycline-based regimens, the 25-year cumulative risks of heart failure after radiotherapy were 4.4%, 6.2%, and 13.3% with 0–15 Gy, 16–20 Gy, and ≥ 21 Gy, respectively, appearing to be dose dependent [77].
(iii) Valvular heart disease
  Radiotherapy can cause thickening, fibrosis, shortening, and calcification of the leaflets and perivalvular tissues. These findings are more predominant in the left-sided than the right-sided valves and are probably related to pressure overload. Radiation-induced valvular disease can be differentiated from rheumatic valve disease by the lesser degree of degeneration of the leaflet tips and commissures and the presence of calcification of the ascending aorta and annulus. Aortomitral curtain thickening/calcification is a hallmark of previous heart irradiation and its extent is associated with mortality [78]. Valvular regurgitation is more commonly encountered than valvular stenosis because of shortening of the valve. Stenotic lesions more often involve the aortic valve. The reported incidence of clinically significant valvular disease is about 1% at 10 years, 5% at 15 years, and 6% at 20 years after radiation exposure. The incidence at 20 years after radiotherapy of mild aortic regurgitation is about 45%, moderate or severe aortic regurgitation about 15%, aortic stenosis about 16%, mild mitral regurgitation about 48%, and mild pulmonary regurgitation about 12% [74]. If surgery is considered in patients with severe valvular disease, transcatheter aortic valve implantation (TAVI) may be preferred for aortic stenosis due to the high risks associated with open heart surgery, such as mediastinal adhesions, high risk pulmonary findings, or calcification of the ascending aorta [73], 79
(iv) Coronary artery disease
  Radiation-induced coronary artery disease develops due to arteriosclerosis accelerated by vascular endothelial injury. It manifests 15–20 years after radiotherapy and is more likely to occur in younger patients [80]. Coronary artery disease is reported to occur in 10% of patients with Hodgkin lymphoma at 20 years after radiation [81].
  Concomitant atherosclerotic risk factors further enhance the development of the disease. In patients with left-sided breast cancer, who undergo left-side chest irradiation, the left coronary main trunk and proximal segments are typically involved [74]. Open heart surgery is high risk, and catheterization for radiation-induced coronary artery stenosis is associated with more restenosis than typical atherosclerotic lesions. Therefore, careful consideration is required for treatment indications.
(v) Carotid artery disease
  As with coronary artery disease, vascular endothelial injury can be induced in vessels within the radiation field, accelerating the progression of arteriosclerosis. Radiotherapy-induced lesions are more extensive, involving atypical areas of carotid segments [74]. A total of 7% of survivors of Hodgkin lymphoma treated with radiation therapy developed carotid and/or subclavian artery disease 20 years after radiotherapy [81]. The relative risk of stroke was 5.6 in patients with head and neck tumors treated with radiotherapy [82]. Therefore, patients receiving radiation to the head and neck require a follow-up for carotid artery disease.

### Follow-up after radiotherapy (Fig. 2)

**Fig. 2:**
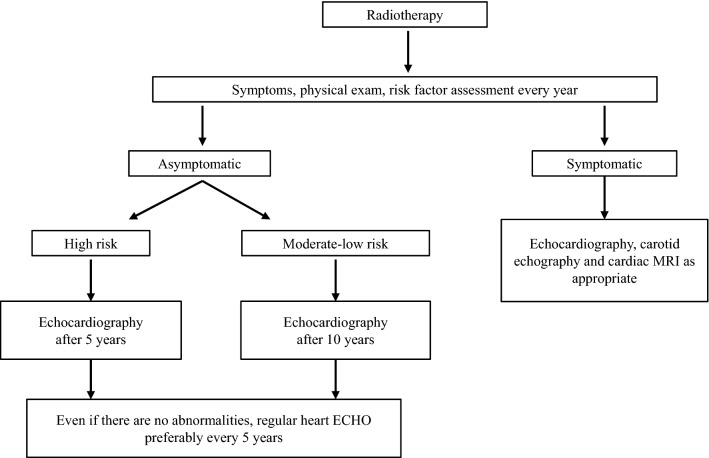
Echocardiographic follow-up after radiotherapy

It is not necessary to perform echocardiography routinely before and during radiotherapy.

As described above, since radiation-induced cardiac damage often becomes overt after several years or even more than a decade after radiation, it is important to perform regular physical examination and risk factor assessment. For patients at high risk, evaluation focusing on coronary lesions should be initiated around 5 years after radiotherapy. Valvular disease becomes apparent later than coronary arterial disease and requires long-term echocardiographic follow-up.

Although regular follow-up is desirable after radiotherapy, there is a lack of evidence regarding its frequency and evaluation parameters, and further accumulation of new findings is awaited.

## Considerations in practical application

### Echocardiographic parameters and measurement errors

Although the development of various automatic measurement techniques has been advancing, the majority of echocardiographic measurements rely on manual measurement and tracing, and the measurement accuracy of parameters depends, to a varying degree, on the examiner's experience and skills. Since the measurement accuracy also depends on the image quality, the reproducibility decreases in cases where images of optimal quality cannot be obtained. The apical approach is difficult in some patients, particularly in those who have undergone left-sided mastectomy. Of note, the reproducibility study of LVEF measurements reported intra- and inter-examiner variability of approximately 5–10%.[83, 84]. GLS measurements also have intra- and inter-examiner variability of approximately 5% [85]. Most of these data were obtained from center hospitals where examiners have adequate echocardiographic experiences and skills. The same reproducibility may not be ensured in community hospitals and clinics. Changes in LVEF and GLS measurements are key factors in making a treatment decision for patients with cancer. Here, we describe quality control and reports of measurements that physicians and sonographers performing echocardiography should be aware of providing safe and appropriate chemotherapy to patients with cancer.

### Quality control in the echocardiography laboratory (Table 10)

**Table 10:** Recommendations for management and maintenance of equipment and quality control in echocardiography laboratories

Class I
Echocardiography laboratory and equipment should be maintained and inspected in accordance with the relevant guidelines
The previous and baseline measurements and images should be reviewed prior to the examination in patients who have previously undergone echocardiography
Echocardiographic measurements should be examined by a well-experienced examiner to ensure agreement with visual assessment
Class IIa
Routine verification of intra- and inter-examiner variability of measurements within the laboratory is recommended to ensure the quality control
Equipment should be adequately maintained to ensure that the images and videos of echocardiography are stored in the storage server and accessed for reference or re-measurement as needed

First, in order for the echocardiographic machines to properly work and perform measurement, it is necessary to carry out maintenance and inspection of the echocardiography laboratory and equipment in accordance with the relevant guideline [86]. To ensure the accuracy of echocardiographic measurements, it is recommended to verify intra- and inter-examiner variability for LVEF and GLS measurements at least once a year to ensure quality control in the laboratory. Above all, the education of the staff who perform echocardiography is of utmost importance for quality control of echocardiographic measurements. Well-experienced staff, such as an sonographer certified by the Japanese Society of Echocardiography and an echocardiologist, should educate inexperienced staff to perform echocardiography uniformly with good accuracy. In addition, equipment should be adequately maintained to ensure that the still images and videos of echocardiography are stored in the storage server and are accessible at any time for reference or re-measurement as needed.

### Considerations for echocardiographic measurements (Table 10)

To make an appropriate treatment decision, it is necessary to minimize the measurement errors of echocardiographic parameters as much as possible. To provide proper measurements, echocardiography should be performed with the following considerations:

- (a) Review of previous and baseline measurements
  In patients who have previously undergone echocardiography, the previous and baseline measurements and images should be reviewed prior to echocardiography (Class I). For this reason, baseline still and motion images should always be recorded. If a well-experienced examiner considers that the previous measurements are not valid compared to the image, they should be re-measured and the examiner should contact the attending physician.
- (b) Measurements by the same examiner
  If possible, the same examiner should be responsible for repeated examinations of the same patient. This does not apply if it is impracticable due to personnel allocation.
- (c) Measurements with the same echocardiographic machine
  Inter-vendor variability of echocardiographic measurements has been reported, particularly for GLS measurements. Standardization has been advanced by a task force led by ASE and EACVI, [85] which has reduced the inter-vendor variability of echocardiographic measurements [87]. However, it is desirable that echocardiographic machines from the same manufacturer be used for the same patient as far as possible. However, this does not apply if it is difficult from the viewpoint of resources for each laboratory.
- (d) Validation of measurements
  Echocardiographic measurements should be assessed by a well-experienced examiner to ensure agreement with visual assessment (Class I). If possible, apart from the examiner, an echocardiologist or certified sonographer should examine the validity of the measurements. In patients who have previously undergone echocardiography, changes in echocardiographic measurements should be examined to ensure agreement with visual changes on the image.

### For deciding appropriate treatment

If there are significant changes in echocardiographic measurements that may lead alterations in chemotherapy, or if there is any doubt about the accuracy of the measurements, the validity of the measurements should be examined by an echocardiologist or certified sonographer as appropriate. If their validity is doubtful, re-measurement or evaluation using other modalities should be performed.

### Future directions

Advances in echocardiography have allowed the automatic analysis of various parameters, and it is expected that the reproducibility will be improved by using automated measurement. It was recently reported that the use of artificial intelligence (AI) improves the accuracy of echocardiographic measurements [88]. In the future, with the use of such technologies, echocardiographic measurements are expected to be performed with good accuracy and excellent reproducibility even by inexperienced examiners.

## Appendix

See Tables

**Table 11:** Anthracycline-based regimens by disease and adriamycin equivalents

	Anthracycline-based regimen	Administration and dose of anthracycline	Adriamycin equivalents at maximum dose
Malignant lymphoma	CHOP	DXR 50 mg/m^2^ × 6–8	400 mg/m^2^
	R-CHOP	DXR 50 mg/m^2^ × 6–8	400 mg/m^2^
	THP-COP	THP 50 mg/m^2^ × 6–8	248 mg/m^2^
	R-THP-COP	THP 50 mg/m^2^ × 6–8	248 mg/m^2^
Breast cancer	EC (epirubicin + cyclophosphamide)	EPI 90 mg/m^2^ × 4	240 mg/m^2^
	ddAC (doxorubicin + cyclophosphamide)	DXR 60 mg/m^2^ × 4	240 mg/m^2^
	AC (doxorubicin + cyclophosphamide)	DXR 60 mg/m^2^ × 4	240 mg/m^2^
	TAC (docetaxel + doxorubicin + cyclophosphamide)	DXR 50 mg/m^2^ × 6	300 mg/m^2^
	FEC (not used currently)	EPI 100 mg/m^2^ × 4 − 9	594 mg/m^2^
Sarcoma (Gynecologic/orthopedic field, etc.)	DXR alone (doxorubicin/adriamycin) (first line)	DXR 75 mg/m^2^ × 6	450 mg/m^2^
Uterine corpus cancer (Recurrent uterine corpus cancer after TC failure)	AP (doxorubicin + cisplatin)	DXR 60 mg/m^2^ × ○	
Recurrent ovarian cancer (Platinum resistant)	Doxil (PLD)	PLD 40 mg/m^2^ × ○	

○: When used for recurrent cancer, DXR is often not administered at the maximum dose in patients with PD by CT assessment or because of adverse reactions. DXR, ADM and ADR, doxorubicin (adriamycin). PLD, doxorubicin hydrochloride liposomal preparation (Doxil): same potency as doxorubicin. EPI, epirubicin (Farmorubicin): Cardiotoxicity is 0.66 times higher than the same dose of doxorubicin. THP, pirarubicin (Therarubicin): Cardiotoxicity is 0.62 times higher than the same dose of doxorubicin

11,

**Table 12:** Description of follow-up echocardiography in the guidance for proper use of anticancer agent

	Brand name	Non-proprietary name (abbreviation)	Description in the guide for proper use
Anti-HER2 (ERBB2) antibody	Herceptin	Trastuzumab	Before treatment and every 3 months during treatment
	Kadcyla	Trastuzumab emtansine	Not described
	Perjeta	Pertuzumab	As per the guide for Herceptin, as used in combination with Herceptin
Molecular-targeting agents other than HER2 (primary target)			
VEGFR	Avastin	Bevacizumab	Before treatment for breast cancer
VEGFR, PDGFR, c-Kit	Votrient	Pazopanib	Mandatory before treatment and at 1 and 3 months are “as clinically indicated.”
VEGFR, FEFR, RET, KIT, PDGFR	Lemvima	Pazopanib	Before treatment
VEGFR, PDGFR, c-kit	Sutent	Sunitinib	Before treatment, at 1 month after the first dose, and at 1 month after every dose
Raf, VEGFR, PDGFR, RET	Nexavar	Sorafenib	Not described
VEGFR	Inlyta	Axitinib	Before treatment, 43 days after Day 1 of Cycle 2, afterward, every 2 cycles
VEGFR	Cyramza	Ramucirumab	Not described
VEGFR, TIE, PDGFR, FGFR, KIT, RET, RAF-1, BRAF	Stivarga	Regorafenib	Not described
bcr-Abl, Abl, PDGFR, KIT	Gleevec/Glivec	Imatinib	Not described
EGFR, HER2	Tykerb	Lapatinib	As appropriate
EGFR	Tagrisso	Osimertinib	Not described
bcr-Abl, Abl, PDGFR, KIT	Tasigna	Nilotinib	Not described
bcr-Abl, Abl, PDGFR, KIT, Src family	Sprycel	Dasatinib	Not described
EGFR	Iressa	Gefitinib	Not described
EGFR	Tarceva	Erlotinib	Not described
EGFR	Erbitux	Cetuximab	Not described
BRAF	Tafinlar	Dabrafenib	Before treatment, at Weeks 3, 12, and 24, afterward every 12 weeks
BRAF	Braftovi	Encorafenib	Not described. As per the guide for Mektovi, when used in combination with Mektovi
MEK	Mekinist	Trametinib	Before treatment, at Weeks 3, 12, and 24, and afterward every 12 weeks
MEK	Mektovi	Binimetinib	Before treatment, at Days 29, 57, 141, 225, 253, and afterward every 28 days
Immune checkpoint inhibitors			
Anti CTLA-4 antibodies	Yervoy	Ipilimumab	Not described
Anti PD-1 antibodies	Opdivo	Nivolumab	Not described
	**Keytruda**	Pembrolizumab	Not described
Anti PD-L1 antibodies	Bavencio	Avelumab	Before treatment and every 2 cycles
	Tecentriq	Atezolizumab	Before treatment and at Cycle 2
	Imifinzi	Durvalumab	Not described

*HER2* human epidermal growth factor receptor 2, *VEGFR* vascular endothelial growth factor receptor, *PDGFR* platelet-derived growth factor receptor, *c-Kit* stem cell factor receptor, *FEFR* fibroblast growth factor, *BRAF* v-raf murine sarcoma viral oncogene homolog B1, *EGFR* epidermal growth factor receptor, *CTLA-4* cytotoxic T-lymphocyte antigen 4, *PD-1* programmed cell death protein 1, *PD-L1* programmed death-ligand 1

^12^.
